# Hepatocellular Carcinoma Confined to the Bile Duct without Hepatic Mass or Jaundice: A Case Report

**DOI:** 10.70352/scrj.cr.25-0429

**Published:** 2025-10-30

**Authors:** Yuto Aoki, Masato Yoshioka, Yohei Kaneya, Kazuhiko Endo, Ryo Ga, Mampei Kawashima, Toshiyuki Irie, Junji Ueda, Tetsuya Shimizu, Youichi Kawano, Yukio Oshiro, Yoshiharu Nakamura, Hiroshi Yoshida

**Affiliations:** 1 Department of Gastroenterological Surgery, Nippon Medical School Chiba Hokusoh Hospital, Inzai, Chiba, Japan; 2 Department of Gastroenterological Surgery, Nippon Medical School Musashikosugi Hospital, Kawasaki, Kanagawa, Japan; 3 Yosei Clinic, Tokyo, Japan; 4 Department of Gastroenterological Surgery, Nippon Medical School, Tokyo, Japan

**Keywords:** differential diagnosis, hepatectomy, hepatocellular carcinoma, intraductal tumor growth, intraductal growth, intrahepatic bile duct

## Abstract

**INTRODUCTION:**

Hepatocellular carcinoma (HCC) presents as a hepatic mass and may involve vascular invasion or extrahepatic spread. However, intraductal growth within the intrahepatic bile duct is rare and is often associated with obstructive jaundice. HCCs confined to the intrahepatic bile duct without detectable hepatic mass or jaundice are rare, and diagnosis is often difficult due to clinical and radiological resemblance to perihilar cholangiocarcinoma. Moreover, such cases generally carry a poor prognosis. We report a rare case of HCC that developed exclusively within the intrahepatic bile ducts, without forming a detectable mass in the liver or causing jaundice.

**CASE PRESENTATION:**

A 70-year-old man presented with right hypochondriac pain. Imaging revealed dilation of the intrahepatic bile ducts in the anterior sector and intraductal filling defects, particularly in the intrahepatic bile duct branch of segment 8, without a detectable hepatic mass. Alpha-fetoprotein and Duke pancreatic monoclonal antigen type 2 levels were elevated, whereas prothrombin induced by vitamin K absence-II, carbohydrate antigen 19-9, and carcinoembryonic antigen levels were within normal limits. Perihilar cholangiocarcinoma was suspected based on imaging. Right hepatectomy with extrahepatic bile duct resection was performed after preoperative portal vein embolization. Intraoperative ultrasonography and gross examination revealed no parenchymal mass. Histopathology showed atypical hepatocyte-like cell clusters with pleomorphic nuclei proliferating within the Glisson’s capsule and infiltrating the adjacent liver parenchyma in a trabecular pattern without fibrous capsule formation. Tumor infiltration into the bile duct epithelium was evident. Immunohistochemical staining was positive for HepPar1 and negative for cytokeratin 19, with a 40% Ki-67 labeling index, confirming HCC diagnosis. The patient remains recurrence-free at 2 years and 7 months postoperatively.

**CONCLUSIONS:**

This case highlights a rare presentation of HCC without a hepatic mass or jaundice, confined to the intrahepatic bile duct. Most patients present with obstructive jaundice. Only two other English-language cases have no prior history of primary HCC, no hepatic mass, no jaundice, and disease confined to the bile duct. Although bile duct-invading HCC is generally associated with poor prognosis, our case suggests that early surgical intervention may lead to favorable long-term outcomes in select patients.

## Abbreviations

AFP
: alpha-fetoprotein
CA19-9
: carbohydrate antigen 19-9
CEA
: carcinoembryonic antigen
DIC-CT
: drip-infusion cholangiography CT
DUPAN-2
: Duke pancreatic monoclonal antigen type 2
EOB-MRI
: gadolinium-ethoxybenzyl-diethylenetriamine pentaacetic acid–enhanced magnetic resonance imaging
FDG
: fluorodeoxyglucose
FLR
: future liver remnant
HCC
: hepatocellular carcinoma
HCV
: hepatitis C virus
KICG
: kinetic indocyanine green elimination rate constant
MRCP
: magnetic resonance cholangiopancreatography
PIVKA-II
: prothrombin induced by vitamin K absence or antagonist-II
US
: ultrasonography

## INTRODUCTION

HCC usually presents as a liver mass that spreads through intrahepatic vascular invasion, which occurs in 15%–57% of surgically resected or transplanted cases.^1)^ By contrast, intraductal extension is relatively uncommon, observed in only 2.3%–13% of surgical and autopsy cases,^2,3)^ and is associated with poor prognosis.^4)^ Patients with intraductal extension often present with obstructive jaundice and are frequently misdiagnosed as having cholangiocarcinoma.

A few reports have described HCC with intraductal involvement in the absence of a liver mass; however, such cases are extremely rare and diagnostically challenging. Here, we present a rare case of primary HCC that manifested solely as an intraductal lesion, with no detectable hepatic mass or jaundice. Notably, the patient achieved long-term recurrence-free survival following surgery, suggesting that early intervention may offer favorable outcomes in select cases.

## CASE PRESENTATION

**Fig. 1:**
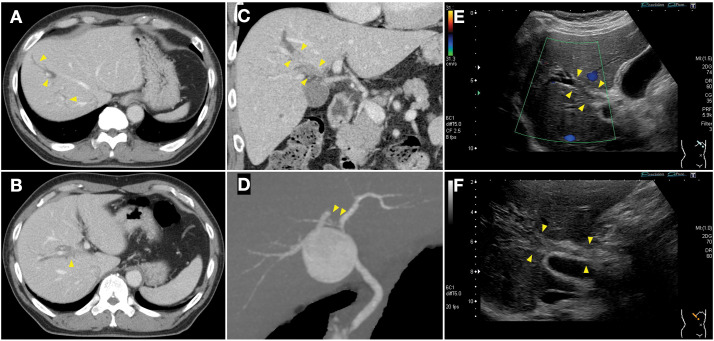
Preoperative imaging studies. (**A**–**C**) Contrast-enhanced CT (CECT). The intrahepatic bile ducts of the anterior section are dilated (**A**), and solid components fill the right hepatic duct and bile duct at the origin of segment B8 (**B**). Coronal reconstructed CECT clearly showing the intraductal lesion and upstream bile duct dilatation (**C**). (**D**) Drip infusion cholangiographic CT. A filling defect can be observed in the right hepatic duct; however, the bile ducts in the anterior section are not visualized. (**E**, **F**) Abdominal ultrasonography. Solid components can also be observed within the right hepatic and bile ducts at the origin of B8, with peripheral intrahepatic bile duct dilation. Arrowheads indicate the location of the intraductal lesion in all panels.

A 70-year-old man presented to the emergency department with pain in the right hypochondrium. Non-contrast abdominal CT revealed localized dilation of the intrahepatic bile ducts, and the patient was referred to our hospital for further evaluation. Contrast-enhanced CT and abdominal US revealed dilated bile ducts in the anterior section and solid components occupying the right hepatic duct and the bile duct branch of segment 8. Coronal reconstructed CT clearly demonstrated the intraductal lesion and upstream bile duct dilatation. DIC-CT showed absence of contrast filling in the B7–8 and common bile duct stump branches, whereas other segments, including B6 and the left hepatic ducts, were normally opacified, with no evidence of choledocholithiasis (**Fig. 1**).

**Fig. 2:**
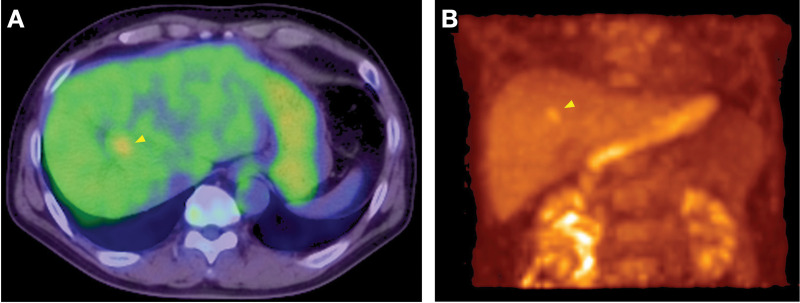
Fluorodeoxyglucose (FDG)-PET/CT findings. (**A**) FDG-PET/CT showing abnormal uptake along the intrahepatic bile duct from segment 8 to the hepatic hilum (SUVmax 3.6–4.1). (**B**) Maximum intensity projection image confirming absence of lymph node or distant metastasis. Arrowheads indicate the location of the intraductal lesion in all panels.

FDG-PET demonstrated abnormal uptake along the intrahepatic bile duct from segment 8 to the hepatic hilum (SUVmax 3.6–4.1), without evidence of lymph node or distant metastasis (**Fig. 2**). EOB-MRI, MRCP, and endoscopic US were not performed.

Laboratory investigations indicated normal CA19-9 and CEA levels, with elevated AFP and DUPAN-2. PIVKA-II levels were within normal limits (**Table 1**). Serological tests revealed positivity for anti-HCV antibody, while hepatitis B surface antigen and hepatitis B core antibody were negative.

**Table 1:** Laboratory findings

Parameter	Result	Unit
WBC	10600	/µL
RBC	4.67	106/µL
Hb	15.4	g/dL
Ht	45.6	%
Plt	318	103/µL
TP	7.7	g/dL
Alb	4.4	g/dL
T-Bil	1.31	mg/dL
D-Bil	0.5	mg/dL
AST	67	U/L
ALT	73	U/L
ALP	648	U/L
GGT	859	U/L
CRP	1.50	mg/dL
PT%	109.1	%
PT-INR	0.96	
APTT	29.3	sec.
D dimer	0.7	µg/mL
CEA	4.4	ng/mL
CA19-9	12.5	U/mL
DUPAN-2	701	U/mL
AFP	61.9	ng/mL
PIVKA-II	27.2	mAU/mL
ICGR15	5.6	%
KICG	0.192	

AFP, alpha-fetoprotein; Alb, albumin; ALP, alkaline phosphatase; ALT, alanine aminotransferase; APTT, activated partial thromboplastin time; AST, aspartate aminotransferase; CA19-9, carbohydrate antigen 19-9; CEA, carcinoembryonic antigen; CRP, C-reactive protein; D-Bil, direct bilirubin; DUPAN-2, Duke pancreatic monoclonal antigen type 2; GGT, gamma-glutamyl transferase; Hb, hemoglobin; Ht, hematocrit; ICGR15, indocyanine green retention rate at 15 min; KICG, kinetic indocyanine green elimination rate constant; PIVKA-II, prothrombin induced by vitamin K absence or antagonist-II; Plt, platelet count; PT%, prothrombin time – percent activity; PT-INR, prothrombin time-international normalized ratio; RBC, red blood cell; T-Bil, total bilirubin; TP, total protein; WBC, white blood cell

**Fig. 3:**
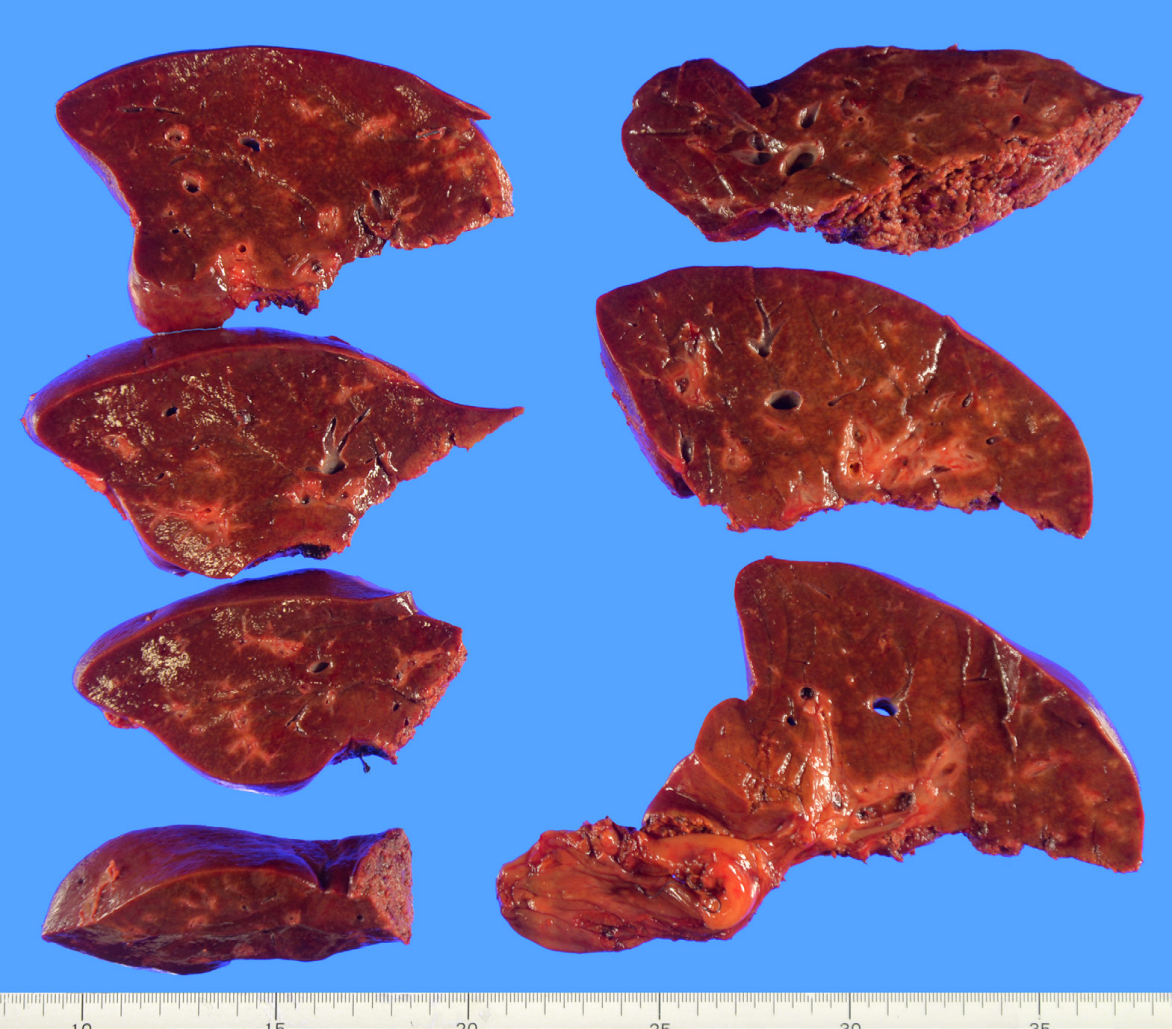
Resected specimen. Macroscopic examination of the resected liver shows no detectable tumor in the hepatic parenchyma.

Imaging and intraoperative findings confirmed tumor involvement of the right hepatic duct and the bile duct branch of segment 8, resulting in widespread bile duct dilatation throughout the right anterior sector. Complete oncological clearance and an adequate surgical margin required right hepatectomy with extrahepatic bile duct resection. Preoperative CT volumetry showed an FLR ratio of 32%, which was considered insufficient. Therefore, right portal vein embolization was performed via the ileocecal vein. After embolization, the FLR increased to 41.7%, with an estimated remnant KICG value (Krem) of 0.0767, deemed acceptable for safe resection. The patient subsequently underwent right hepatectomy and extrahepatic bile duct resection with Roux-en-Y hepaticojejunostomy. Intraoperative US revealed no hepatic masses; however, bile duct stenosis was observed. Gross examination of the resected specimen showed no distinct tumors within the liver parenchyma (**Fig. 3**).

Histopathological examination revealed that the tumor was mainly located within the lumen of the intrahepatic bile duct and exhibited poorly differentiated hepatocellular carcinoma morphology. Although no gross hepatic parenchymal mass was identified, microscopic foci of tumor infiltration into the adjacent liver parenchyma were observed in a trabecular growth pattern without capsule or septum formation. Importantly, the tumor was not continuous with the bile duct epithelium but proliferated within the bile duct lumen.

**Fig. 4:**
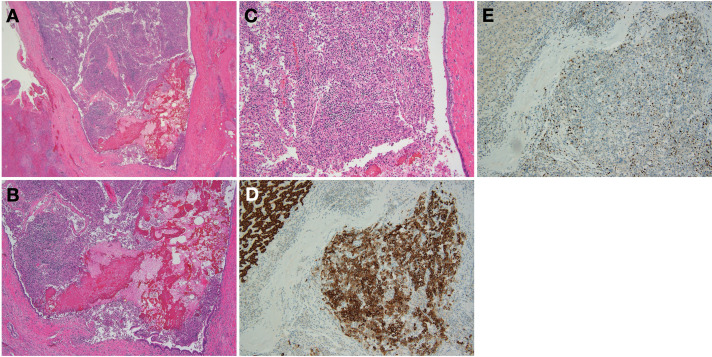
Histopathological findings. (**A**) Clusters of atypical cells are observed within the bile duct lumen (hematoxylin and eosin, ×20). (**B**, **C**) Atypical cells with pleomorphic polygonal morphology resembling hepatocyte-like cells. Cells infiltrate the surrounding hepatic parenchyma in a sponge-like trabecular pattern without forming a capsule or fibrous septum (hematoxylin and eosin, (**B**) ×40; (**C**) ×100). (**D**) Immunohistochemistry shows strong positivity for HepPar1 (×100). (**E**) The Ki-67 labeling index is approximately 40% (×100). These findings support the diagnosis of hepatocellular carcinoma arising within the bile duct lumen.

Elastic Van Gieson staining confirmed vascular invasion, with tumor cells identified in small branches of the portal vein (Vp1) and hepatic vein (Vv1), whereas no hepatic arterial invasion (Va0) was detected. The background liver showed mild fibrosis (F1) and moderate inflammatory activity (A2), without significant steatosis or cirrhosis. Immunohistochemically, the tumor cells were positive for HepPar1 and negative for CK19, with a Ki-67 labeling index of approximately 40%, confirming the diagnosis of HCC (**Fig. 4**).

The patient developed an anastomotic leak at the jejunojejunostomy site postoperatively, necessitating surgical repair. The patient recovered without further complications and was discharged on POD 27 following the initial surgery. The patient remained recurrence-free at 2 years and 7 months after surgery, after which follow-up was discontinued because of an unrelated condition.

## DISCUSSION

HCC typically presents as a mass within the liver and frequently invades the portal or hepatic veins. By contrast, intraductal growth is relatively rare and considered as a poor prognostic factor.^4)^ Among such cases, some are classified as icteric-type HCC, characterized by bile duct invasion leading to obstructive jaundice.^5)^ In previously reported cases of HCC with bile duct involvement, including icteric-type HCC, a distinct mass lesion is typically present in the liver.

However, in the present case, the tumor was limited to the intrahepatic bile duct, with no identifiable liver mass or jaundice. This may represent an early stage of icteric-type HCC in which only regional bile duct obstruction is present without involvement of the hepatic or common bile ducts. Alternatively, a small intrahepatic tumor may regress or become undetectable at the time of surgery, leaving only an intrahepatic component. In our patient, it is also possible that a microscopic lesion was present but remained below the detection threshold for imaging and histopathology. Another possibility, although speculative, is that the tumor originated from ectopic hepatocytes located within the bile duct mucosa, a phenomenon previously reported in rare pathological studies.^6,7)^

The diagnosis of HCC in the absence of a detectable hepatic mass is extremely challenging. Most reported cases of such presentation initially manifest with obstructive jaundice.^6–14)^ Several cases of HCC presenting with bile duct involvement but without a detectable intrahepatic mass have been previously reported (**Table 2**). While most of these cases manifested with obstructive jaundice, few were reported without jaundice. True primary HCCs confined to the bile duct without intrahepatic tumors were rare, with other cases representing recurrent disease. Although a few cases of HCC confined solely to the bile duct without jaundice have been described, these include both primary and recurrent tumors.^15)^ Reports of primary HCC presenting solely as an intraductal lesion without a hepatic mass or jaundice are exceedingly rare. To the best of our knowledge, only 2 such cases fulfilling all of the following criteria have been documented: (1) a first-time pathological diagnosis of HCC (i.e., no recurrence), (2) no identifiable hepatic mass on imaging or histopathology, (3) disease confined to the bile duct, and (4) no clinical or laboratory evidence of jaundice. One such case was included in a Japanese report by Kondo et al.,^16)^ who described 2 patients with bile duct-invading HCC without detectable hepatic tumors. However, only one of these patients met all the above criteria, whereas the other presented jaundice.

**Table 2:** Reported cases of HCC with bile duct involvement but without detectable intrahepatic mass

Author, Year	Age	Sex	Chief complaint	Jaundice	Hepatic mass	Risk Factor	T-Bil (mg/dL)	AFP (ng/mL)	PIVKA-II (mAU/mL)	Tumor location	Initial/Recurrence	Preoperative diagnosis	Pathological diagnosis	Outcome (after liver resection)
Kondo, 2003^16)^ Case 1	55	Female	General fatigue	No	Absent	None	1	<5	ND	Right anterior intrahepatic bile duct	Initial	Bile duct carcinoma	Poorly differentiated HCC	Recurrence at 4 m and died at 5 m
Kondo, 2003^16)^ Case 2	35	Male	Jaundice and epigastric pain	Yes	Absent	HBV	1.9	6	9	Left hepatic duct	Initial	Bile duct carcinoma	Poorly differentiated HCC	Recurrence at 8 m and died at 3 y
Makino, 2006^8)^	70	Male	Jaundice	Yes	Absent	HCV	ND	9	19200	Common bile duct–left hepatic duct	Initial	Bile duct carcinoma	Well-differentiated HCC	Recurrence-free at 1 y
Chang, 2010^10)^	54	Male	Jaundice and right upper abdominal pain	Yes	Absent	HBV	12.6	Normal range	ND	Common bile duct	Initial	Bile duct carcinoma	HCC	Recurrence at 2 w, died at 1 m
Abe, 2012^15)^	72	Female	None (routine checkup after HCC treatment)	No	Absent	HCV	ND	2.9	13	Right anterior intrahepatic bile duct	Recurrence	Recurrent HCC or bile duct carcinoma	Poorly differentiated HCC (HCC recurrence in bile duct)	Recurrence-free at 1 y
Alshati, 2019^14)^	40	Male	Jaundice, upper quadrant pain and weight loss	Yes	Absent	HBV	3.2	73.95	ND	Left hepatic duct	Initial	HCC (biopsy via ERCP)	Poorly differentiated HCC	ND
Nakashima, 2025^17)^	74	Male	None (routine checkup after HCV treatment)	No	Absent	HCV	Normal range	Normal range	512	B2–B3	Initial	Intrahepatic cholangiocarcinoma	HCC	Recurrence at 6 m
Present case (Aoki, 2025)	70	Male	Epigastric pain	No	Absent	HCV	1.31	109.1	27.2	Right hepatic duct–B8	Initial	Perihilar cholangiocarcinoma	Poorly differentiated HCC	Recurrence-free at 2 y 7 m

This table summarizes representative cases cited in the present report, including those with or without obstructive jaundice. It does not aim to comprehensively cover all reported cases of icteric-type HCC, but rather highlights those most relevant to the discussion.

AFP, alpha-fetoprotein; ERCP, endoscopic retrograde cholangiopancreatography; HBV, hepatitis B virus; HCC, hepatocellular carcinoma; HCV, hepatitis C virus; m, month; ND, not described; PIVKA-II, protein induced by vitamin K absence or antagonist-II; T-Bil, total bilirubin; w, week; y, year

A comparable case was recently reported by Nakashima et al.^17)^ in 2025, who described an HCC confined to the intrahepatic bile duct without a hepatic mass or jaundice. Despite comprehensive preoperative endoscopic evaluations including cholangioscopy and biopsy, a definitive diagnosis was not established until after surgery. Notably, the patient developed intraductal recurrence within 6 months postoperatively.

A comparable case reported by Kondo et al.^16)^ also lacked a detectable hepatic mass and jaundice; however, the patient experienced early intrahepatic recurrence and died within 5 months of surgery. By contrast, our patient remained recurrence-free for 2 years and 7 months postoperatively despite the absence of preoperative histological confirmation. These differing clinical courses suggest that intraductal HCC without hepatic parenchymal involvement may exhibit variable biological behavior and that early surgical intervention may contribute to a favorable prognosis in select cases.

Although the number of reported cases is limited, further case accumulation may help clarify whether this presentation, characterized by the absence of a hepatic mass and jaundice, represents a distinct prognostic subgroup compared with the more common bile duct-invasive HCCs associated with intrahepatic tumors and/or obstructive jaundice.

Endoscopic procedures such as cholangioscopy or bile duct biopsy also play a crucial role in establishing the diagnosis.^18)^ However, in our case, preoperative endoscopic evaluation was not feasible, and the patient underwent surgery under a presumptive diagnosis of perihilar cholangiocarcinoma. This highlights the diagnostic challenge of intraductal HCC mimicking bile duct carcinoma in the absence of a detectable hepatic mass.

Minimally invasive liver surgery, including robotic liver resection, has recently gained attention owing to its applicability in complex resections, including those involving the biliary tract. Although not applied in this case, such approaches, particularly robot-assisted hepatectomy, may offer a future surgical option for complex biliary cases where the preoperative diagnosis is inconclusive.^19)^

## CONCLUSIONS

We present a rare case of HCC manifesting solely as an intraductal lesion with no detectable hepatic mass, jaundice, or prior history of HCC. The clinical and radiological findings closely resembled those of perihilar cholangiocarcinoma, highlighting the diagnostic challenges of such cases. Although, in a similar case, early recurrence has been reported,^16,17)^ our patient was recurrence-free for over 2 years and 7 months, suggesting that early surgical intervention may contribute to favorable long-term outcomes. Further case accumulation is essential to better understand prognosis and optimize diagnostic and therapeutic strategies.

These findings emphasize the importance of recognizing intraductal HCC in the differential diagnosis and suggest that prompt surgical intervention before the development of jaundice or a hepatic mass may help to improve long-term outcomes.

## DECLARATIONS

### Availability of data and materials

The dataset supporting the conclusions of this article is included within the article.

## References

[1] Rodríguez-Perálvarez M, Luong TV, Andreana L, et al. A systematic review of microvascular invasion in hepatocellular carcinoma: diagnostic and prognostic variability. Ann Surg Oncol 2013; 20: 325–39. 23149850 10.1245/s10434-012-2513-1

[2] Kojiro M, Kawabata K, Kawano Y, et al. Hepatocellular carcinoma presenting as intrabile duct tumor growth. A clinicopathologic study of 24 cases. Cancer 1982; 49: 2144–7. 6280834 10.1002/1097-0142(19820515)49:10<2144::aid-cncr2820491026>3.0.co;2-o

[3] Nakashima T, Okuda K, Kojiro M, et al. Pathology of hepatocellular carcinoma in Japan: 232 consecutive cases autopsied in ten years. Cancer 1983; 51: 863–77. 6295617 10.1002/1097-0142(19830301)51:5<863::aid-cncr2820510520>3.0.co;2-d

[4] Yang X, Qiu Z, Ran R, et al. Prognostic importance of bile duct invasion in surgical resection with curative intent for hepatocellular carcinoma using PSM analysis. Oncol Lett 2018; 16: 3593–602. 30127966 10.3892/ol.2018.9108 PMC6096155

[5] Lin TY, Chen KM, Chen YR, et al. Icteric type hepatoma. Med Chir Dig 1975; 4: 267–70. 173942

[6] Schmelzle M, Matthaei H, Lehwald N, et al. Extrahepatic intraductal ectopic hepatocellular carcinoma: bile duct filling defect. Hepatobiliary Pancreat Dis Int 2009; 8: 650–2. 20007086

[7] Tsushimi T, Enoki T, Harada E, et al. Ectopic hepatocellular carcinoma arising in the bile duct. J Hepatobiliary Pancreat Surg 2005; 12: 266–8. 15995818 10.1007/s00534-004-0963-y

[8] Makino T, Nakamori S, Kashiwazaki M, et al. An icteric type hepatocellular carcinoma with no detectable tumor in the liver: report of a case. Surg Today 2006; 36: 633–7. 16794800 10.1007/s00595-006-3214-9

[9] Abdullah A, Jenkins-Mosure K, Lewis T, et al. Primary hepatoid carcinoma of the biliary tree: a radiologic mimicker of Klatskin-type tumor. Cancer Imaging 2010; 10: 198–201. 10.1102/1470-7330.2010.0027 PMC2999407 20934950

[10] Chang H, Xu J, Mu Q, et al. Occult hepatocellular carcinoma: a case report of a special icteric-type hepatoma and literature review. Eur J Cancer Care (Engl) 2010; 19: 690–3. 19659667 10.1111/j.1365-2354.2008.01035.x

[11] Long XY, Li YX, Wu W, et al. Diagnosis of bile duct hepatocellular carcinoma thrombus without obvious intrahepatic mass. World J Gastroenterol 2010; 16: 4998–5004. 20954289 10.3748/wjg.v16.i39.4998 PMC2957611

[12] He M, Wang H, Ji F, et al. Diagnosis and therapy of ectopic hepatocellular carcinoma growing in the intrahepatic bile duct. J Coll Physicians Surg Pak 2016; 26: 130–2. 28666506

[13] Philips CA, Paramaguru R, Mahadevan P, et al. Isolated intraductal variant of hepatocellular carcinoma. Case Reports 2017; 2017: bcr-2017-221324. 10.1136/bcr-2017-221324 PMC5624085 28801513

[14] Alshati A, Bellapravalu S, Srinivasan I, et al. Imaging-negative hepatocellular carcinoma presents as an intrabiliary mass. ACG Case Rep J 2019; 6: e00068. 31616745 10.14309/crj.0000000000000068 PMC6658063

[15] Abe T, Kajiyama K, Harimoto N, et al. Intrahepatic bile duct recurrence of hepatocellular carcinoma without a detectable liver tumor. Int J Surg Case Rep 2012; 3: 275–8. 22516418 10.1016/j.ijscr.2012.03.017 PMC3356541

[16] Kondo M, Dono K, Sakon M, et al. Two cases of hepatocellular carcinoma with bile duct invasion, but without a detectable tumor in the liver (in Japanese with English abstract). Jpn J Gastroenterol Surg 2003; 36: 482–7.

[17] Nakashima Y, Hiramatsu K, Fukaya M, et al. A case of hepatocellular carcinoma arising from the intraductal hepatic bile duct without parenchymal lesion. Clin J Gastroenterol 2025; 18: 195–201. 39433708 10.1007/s12328-024-02054-2

[18] Koo CS, Ho KY, Pang YH, et al. Extraction of intra-biliary hepatocellular carcinoma by endoscopic retrograde cholangiopancreatography. BMC Gastroenterol 2020; 20: 408. 33287724 10.1186/s12876-020-01552-0 PMC7720534

[19] Minamimura K, Aoki Y, Kaneya Y, et al. Current status of robotic hepatobiliary and pancreatic surgery. J Nippon Med Sch 2024; 91: 10–9. 38233127 10.1272/jnms.JNMS.2024_91-109

